# Supplementary material for: Complex‐centric proteome profiling by SEC‐SWATH‐MS
Source: Mol Syst Biol. 2019 Jan 14;15(1):e8438. doi: 10.15252/msb.20188438 (PMC6346213; doi:10.15252/msb.20188438)
Supplement: Supplementary file 6 — Dataset EV5 [file MSB-15-e8438-s006.zip › feature_plots_corum/2920.pdf]

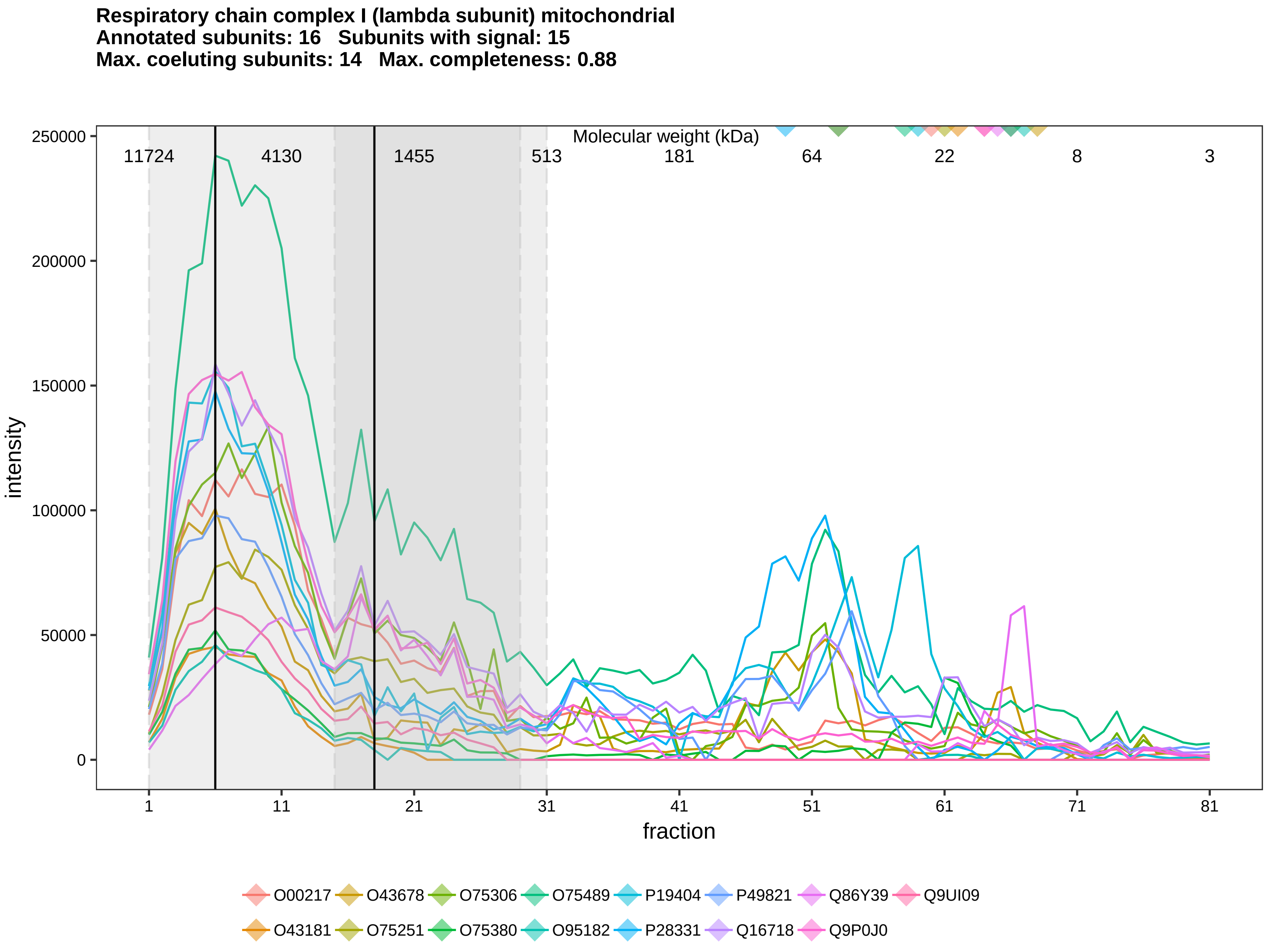

Respiratory chain complex I (lambda subunit) mitochondrial
Annotated subunits: 16 Subunits with signal: 15
Max. coeluting subunits: 14 Max. completeness: 0.88
Molecular weight (kDa)
11724
4130
1455
513
181
64
22
8
3
intensity
250000
200000
150000
100000
50000
0
fraction
1
11
21
31
41
51
61
71
81
O00217
O43678
O75306
O75489
P19404
P49821
Q86Y39
Q9UI09
O43181
O75251
O75380
O95182
P28331
Q16718
Q9P0J0
